# Online Information About Cardiac Neurodevelopment: Mixed Methods Study

**DOI:** 10.2196/70630

**Published:** 2026-07-14

**Authors:** Jesse Boyett Anderson, Gabrielle Chinman, Diego Cisneros, Xiao Zhang, Peter Ferrazzano, Krisjon R Olson

**Affiliations:** 1 Department of Pediatrics, Division of Pediatric Cardiology, University of Wisconsin School of Medicine and Public Health, 600 Highland Avenue, Madison, WI, 53792, United States, 1 6088904258, 1 6088908102; 2 Department of Neurology, UW Health, Madison, WI, United States; 3 David Geffen School of Medicine, University of California, Los Angeles, Los Angeles, CA, United States; 4 Department of Pediatrics, Division of Pediatric Critical Care Medicine, University of Wisconsin School of Medicine and Public Health, Madison, WI, United States

**Keywords:** heart defects, congenital, neurodevelopment, online health information seeking, digital media, pediatric hospital, caregiver, qualitative research, cardiac surgery, quality of life, OHIS

## Abstract

**Background:**

Infants with complex heart disease often have delayed development, learning difficulties, and mental health problems as they grow older. Their parents and other caregivers engage in online health information–seeking behavior to understand and support their children’s health and development.

**Objective:**

In this study, we analyze the presence, nature, and presentation of information about neurodevelopment on the websites of congenital cardiac surgical programs in the United States.

**Methods:**

We used a mixed methods approach, specifically a convergent design. We correlated the presence and presentation of information about neurodevelopment on each program’s website with state-level and program-specific factors extracted from multiple publicly available databases. Quantitative analysis methods included descriptive analyses, Student *t* tests, Pearson chi-square test, and multivariate logistic regression analysis, all performed using SPSS (IBM Corp). Qualitative methods included both inductive and deductive content analysis.

**Results:**

Only 39% (50/129) of programs provided any information about neurodevelopment online. High surgical volume correlated with the presence of online information (*P*<.001). Websites were written at an average 7th to 10th grade reading level, and fewer than 5% of websites had information in a language other than English. Two semantic clusters of website format, content, and element selection were identified, reflecting distinct approaches to adult learners. Program websites clustered into “sage on the stage” and “guide on the side” formats. Few websites incorporated features caregivers previously identified as useful, with only 6% including the four most helpful features.

**Conclusions:**

We highlight the paucity of accessible caregiver-oriented information about neurodevelopment on congenital cardiac surgical programs’ websites in the United States and characterize two primary website models. The lack of information may negatively impact caregiver understanding of and engagement with neurodevelopmental services for their child with a congenital heart defect. Future research should explore the impact of each website model on caregiver understanding of and engagement with neurodevelopmental services, as well as the generalizability of these findings to other domains of pediatric subspecialty care.

## Introduction

Congenital heart disease (CHD) is the most common type of birth defect, affecting nearly 1% of babies born in the United States every year [1]. Survival has improved with advances in medical and surgical management [2]. As survival has improved, neurodevelopmental impairments have emerged as the predominant morbidity for neonatal heart surgery. Such impairments in gross motor skills, executive functioning, and mental health are considered one of the primary determinants of quality of life across the patient’s lifespan [3-5]. Many of these impairments are amenable to treatment; however, many children do not receive appropriate screening, with parent and other caregiver understanding one likely barrier to screening [6,7].

Parents and other caregivers (grandparents, foster parents, etc) of children with CHD prefer more information than pediatric cardiologists provide, particularly about their child’s future quality of life [8]. As with other patients and caregivers, they fill their knowledge gaps using online health information-seeking behaviors [9-13]. Websites rival health care professionals as the most common source of information caregivers use to learn about their child’s heart condition (72%‐92% compared with 77%‐92%, respectively), with 30% of those caregivers reporting the use of hospital websites [11,12]. While most parents report searching for information about their child’s cardiac condition (88%), many also search for information about long-term outcomes (73%) and challenges associated with CHD (59%) [11]. A handful of studies have evaluated online information about congenital heart defects, but no previous studies have evaluated congenital cardiac surgical program websites, either overall or for information specifically related to the neurodevelopmental impacts of CHD [14,15]. It is unclear if currently available information is adequate to inform caregiver understanding of the need for and engagement with neurodevelopmental supports for their child.

This study analyzes the information about neurodevelopment available on the websites of congenital cardiac surgical programs in the United States. Given heterogeneity in both congenital cardiac surgical outcomes and health care funding and delivery across the United States, we hypothesized that center-specific factors as well as state-level sociodemographic variables would predict the presence, reliability, and models of information provided on these websites.

## Methods

### Sample Selection

A total sample of all US congenital cardiology surgical programs was defined using the 2021‐2022 Congenital Cardiology Today directory. Given the impact of funding, politics, and values on institutional priorities and behaviors, a database containing program-specific and state-level characteristics of each program was created. Metrics reflecting the size and robustness of each congenital cardiac surgical program, sociodemographic characteristics of the children in the state being served by each program, and each state’s investment in the health and education of children, all extracted from publicly available data, were included in this database (Table 1).

Websites for each congenital cardiac surgical program with information on cardiac neurodevelopment were identified between June and October 2022 using a two-step strategy. First, congenital cardiac surgical program websites were identified. Second, each website was queried for information on neurodevelopment. All web searches described below were conducted by an undergraduate researcher from the Midwest with no prior exposure to the topics of pediatric cardiology or cardiac neurodevelopment.

**Table 1.** Publicly available data sources used to identify program-specific and state-level characteristics.

Data source	Data extracted
Congenital Cardiology Today Directory (2021‐2022)+ program websites	
	Program size (cardiologists [n])
STS^a^ Public Reporting Database 2015‐2018	
	Surgical cases (n)
	STAT^b^ 4+5 cases (%)
	Overall observed/expected mortality
FREIDA^c^ 2022	
	Presence of a pediatrics residency program
	Presence of a pediatric cardiology fellowship program
US Census 2020	
	State population who are BIPOC^d^ (%)
	State population that is Hispanic/Latino (%)
ACS^e^ 2016‐2019	
	State unemployment rate
	Children in the state living in poverty (%)
	Children in the state without insurance (%)
	Children in the state on Medicaid (%)
Kaiser Family Foundation Reporting 2019	
	Whether or not a state had accepted Medicaid expansion
National Center for Education Statistics 2019	
	State-level student-teacher ratio in K-12 classrooms
IDEA^f^ Part B Child Count and Educational Environments Report 2019‐2020	
	Students in K-12 classrooms with an IEP^g^ (%)

a STS: Society of Thoracic Surgeons.

b STAT: Society of Thoracic Surgeons–European Association for Cardio-Thoracic Surgery.

c FREIDA: Fellowship and Residency Interactive Electronic Database.

d BIPOC: biracial or people of color.

e ACS: American Community Survey.

f IDEA: Individuals with Disabilities Education Act.

g IEP: individualized education plan.

### Website Identification

To maximize search results, a two-pronged approach was used to find congenital cardiac surgical program websites. First, the websites listed in the Congenital Cardiology Today directory were visited. If the listed website was specific to the congenital heart program at that institution, the URL was saved and the splash page was saved as HTML. If the listed website was an institutional website not specific to the congenital heart program, the institutional website search bar was queried sequentially for nontechnical keywords: “heart,” “heart defect,” “heart surgery,” “congenital heart defect,” “pediatric cardiology,” and “cardiac defect.” English language terms were chosen as this is the official language of the country in which all programs were located. Approximating the strategy used by individuals searching for health information online, the first 10 results of each search were reviewed [16]. If a congenital heart program website was found, the URL was saved and the splash page was saved as HTML. This search strategy generated a repository of institutional congenital heart program websites.

### Neurodevelopmental Information Identification

#### Overview

Each congenital cardiac surgical program website was then searched for information about neurodevelopment. Once again, a two-pronged approach was used, this time to allow for the evaluation of internal and external search engine optimization. First, all previously identified congenital cardiac surgical program websites were reviewed to identify the presence of any information related to neurodevelopment. Second, a Google (Google LLC) search was performed to identify information about cardiac neurodevelopment on the institutional website. The webpages identified using this process formed the units of analysis for subsequent qualitative analysis.

#### Institutional Website Search

Each congenital cardiology program website, regardless of whether it was identified through the *Congenital Cardiology Today* directory or via a search of the institutional website, was reviewed to identify any information about neurodevelopment. Search terms were preselected and included “development,” “neurodevelopment,” “neuropsychology,” and “brain.” If no mention was found on review of each website, the websites were searched using Command-F and through the site search bar on the institutional website. The first 10 results of each search were reviewed [16]. If information about cardiac neurodevelopment was identified, the URL was saved, the splash page was saved as HTML, and all text and images were scraped and archived in the archived qualitative data analysis software Dedoose (Sociocultural Research Consultants, 2022).

#### Google Search

A Google search for each program’s name as listed in the Congenital Cardiology Today directory, in conjunction with preselected keywords, was performed. To maximize search results, keywords were selected based on the verbiage found on the websites of institutions with program websites and included “neurodevelopment,” “cardiac psychology,” “neurodevelopmental follow-up,” and “cardiac neurodevelopment.” The first 10 results of each search were reviewed [16]. If a program website was found, the URL was saved, the splash page was saved as HTML, and all text and images were archived as described above.

### Mixed Methodology

**Figure 1.**
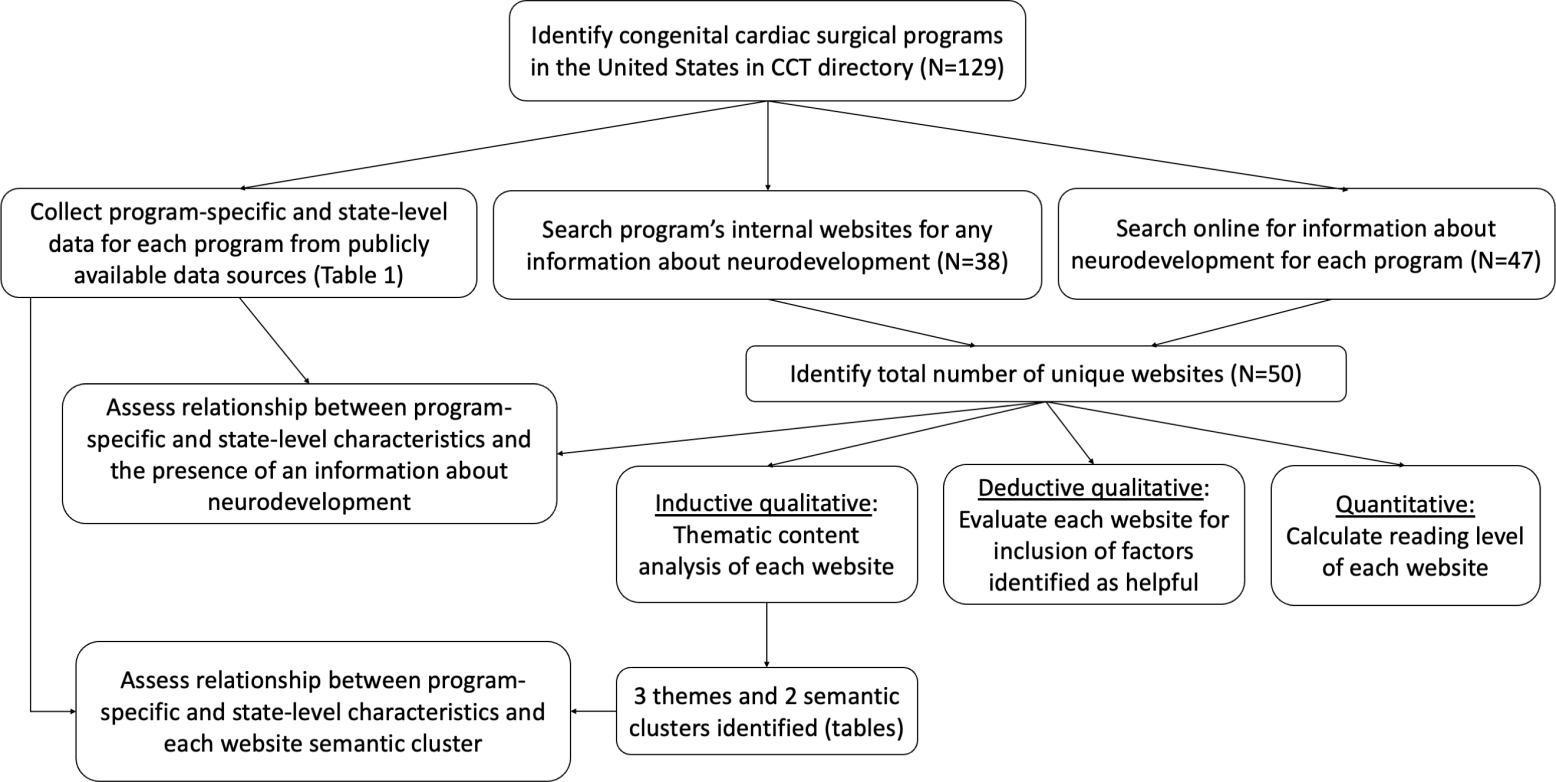
Study design overview. CCT: Congenital Cardiology Today.

A convergent mixed methods design was used with parallel quantitative and qualitative analyses, each informing the other (Figure 1) [17]. The central research question was: “How do congenital cardiac surgical programs in the United States communicate information about cardiac neurodevelopment online?” We also asked the subquestion: “Which program-specific and state-level factors impact how this communication occurs?” Results are reported following the Standards for Reporting Qualitative Research [18].

### Quantitative Analysis

A total population sample requires no inferential statistical analysis because descriptive analysis captures the full population. However, given imperfections in all sampling techniques, univariate and multivariate analysis of program-specific and state-level characteristics correlating with the presence of information about neurodevelopment on congenital cardiac surgical program websites were performed using chi-square tests, *t* tests for the equivalence of the means, and multivariate logistic regression analysis. The program-specific and state-level characteristics are listed in Table 1. For multivariate analysis, surgical programs were binned into even thirds based on surgical volume, with cut points at 125 cases and 270 cases per year, and based on surgical complexity with cut points at <21.75% Society of Thoracic Surgeons–European Association for Cardio-Thoracic Surgery 4+5 cases and >26.75% Society of Thoracic Surgeons–European Association for Cardio-Thoracic Surgery 4+5 cases. Program size was binarized, with larger programs being defined as those with at least 20 cardiologists. Two-sided significance was defined as *P*<.05, and all analyses were performed with SPSS (version 28).

### Qualitative Analysis

#### Inductive Content Analysis

Inductive content analysis of the archived websites was performed between July 2022 and October 2023. This data-driven strategy involves approaching the data without an a priori code book. Instead, a code book is constructed as the data is reviewed in an effort to “remain open to new discoveries” [19]. Codes emerged as the team collaboratively examined the archived websites and created analytic memos documenting their analysis and coding structure. In this fully crossed design, all websites were coded by each coder.

The aims of this analysis were two-fold: (1) to identify semantic clusters based on the types and modalities of information shared on this diverse group of websites; and (2) to situate these clusters within the intersecting geographic, academic, medical, social, and interpersonal spaces occupied by congenital cardiac surgical programs within the United States [20].

#### Positionality and Reflexivity

The four primary coders have expertise in health equity (DC), psychology (GC), medical anthropology (KRO), and pediatric cardiology (JBA). One is the parent of a child with CHD (JBA). One has studied CHD at two heart centers in the Midwest, one in the urban Pacific Northwest, and one in the urban South (KRO). Two were undergraduate students with no previous exposure to CHD at the time of this study (DC and GC). This study grew out of one author’s experience seeking information online about her son’s heart defect and its long-term implications (JBA).

### Deductive Content Analysis

#### Language Accessibility

The readability of each website was calculated using multiple measures, including the Flesch-Kincaid readability test, SMOG (Simplified Measure of Gobbledygook) index, and Gunning Fog [21].

#### Caregiver Utility

Each website was coded for the presence of the top four features preferred by parents of children with CHD [11]. These features include (1) a combination of words, pictures, and videos; (2) descriptions of current research; (3) patient stories; and (4) links to additional websites.

#### Transparency and Reliability

The transparency and reliability of each website were calculated using JAMA (Journal of the American Medical Association) benchmarks, a streamlined tool to assess “the quality of medical information on the internet” [22]. JAMA benchmarks uses four criteria: authorship, attribution, disclosure, and currency. Scores range from 0 to 4.

## Results

### Sample

All 129 congenital cardiac surgical programs in the United States listed in the Congenital Cardiology Today Directory were analyzed (Table 2). Only 50 (39%) programs included information about cardiac neurodevelopment on their program website. This information could be found by searching within the program website for 38 programs and via a Google search for 47 programs.

**Table 2.** Program-specific and state-level characteristics of all US congenital cardiac surgical programs.

Characteristic	Values
Programs, n (%)	
US programs in the Congenital Cardiology Today Directory	129
Neurodevelopmental information findable on Google	47 (36)
Neurodevelopmental information findable on program website	38 (30)
Neurodevelopmental information findable by either method	50 (39)
Program-specific characteristics	
Contributed to STS^a^ database, n (%)	91 (70)
Pediatric residency program, n (%)	106 (82)
Pediatric cardiology fellowship, n (%)	65 (50)
Number of surgical cases (over 4 years), mean (SD)	899 (671)
STAT^b^ 4+5 cases (%), mean (SD)	24 (6)
Observed/expected mortality, mean (SD)	1 (0.5)
State-level characteristics	
BIPOC^c^ (%), mean (SD)	38 (14)
Hispanic/Latino (%), mean (SD)	19 (13)
Unemployment rate, mean (SD)	5 (1)
Children in poverty (%), mean (SD)	18 (4)
Children uninsured, (%) mean (SD)	5 (2)
Children on Medicaid (%), mean (SD)	38 (6
Accepted Medicaid expansion, n (%)	95 (74)
Student to teacher ratio, mean (SD)	16 (3)
Students served by IDEA^d^ (%), mean (SD)	13 (2)

a STS: Society of Thoracic Surgeons.

b STAT: Society of Thoracic Surgeons–European Association for Cardio-Thoracic Surgery.

c BIPOC: biracial or person of color.

d IDEA: Individuals with Disabilities Education Act.

### Quantitative Analysis

Five congenital cardiac surgical program–specific factors, and no state-level factors, correlated with the presence of information about neurodevelopment on surgical program websites (Table 3). Only surgical volume retained significance after multivariate analysis: programs averaging more than 270 surgical cases a year were significantly more likely than lower-volume programs to post information about neurodevelopment on their website (*P*<.001; Table 4).

**Table 3.** Program-specific and state-level characteristics correlated with the presence of online information about neurodevelopment (univariate analysis).

Characteristic	Values (*df*)	*P* value
Program-specific characteristics		
Contributed to STS^c^ database	12.602 (1)^a^	<.001
Pediatric residency program	9.299 (1)^a^	.002
Pediatric cardiology fellowship	27.448 (1)^a^	<.001
Number of surgical cases (over 4 years)	−6.221 (89)^b^	<.001
STAT^f^ 4+5 cases (%)	−2.415 (89)^b^	.009
Observed/expected mortality	1.421 (89)^b^	.08
State-level characteristics		
BIPOC^d^ (%)	0.824 (127)^b^	.21
Hispanic/Latino (%)	1.323 (127)^b^	.09
Unemployment rate	0.249 (127)^b^	.40
Children in poverty (%)	−0.684 (127)^b^	.25
Children uninsured (%)	0.261 (127)^b^	.40
Children on Medicaid (%)	0.876 (127)^b^	.19
Accepted Medicaid expansion	0.332 (1)^a^	.55
Student to teacher ratio	−0.210 (127)^b^	.42
Students served by IDEA^e^ (%)	0.694 (125)^b^	.25

a Chi-squared test used to evaluate binary variables.

b *t* test for the equivalence of the mean used to evaluate continuous variables.

c STS: Society of Thoracic Surgeons.

d BIPOC: biracial or person of color.

e IDEA: Individuals with Disabilities Education Act.

f STAT: Society of Thoracic Surgeons–European Association for Cardio-Thoracic Surgery.

**Table 4.** Factors correlated with the presence of information about neurodevelopment on program websites (multivariate analysis).

Program-specific characteristics	Odds ratio (95% CI)	*P* value
Pediatric Cardiology Fellowship Program present	1.1 (0.30-4.40)	.86
Pediatrics residency program present	11.8 (0.80-178)	.07
Surgical volume^a^		
Lowest tertile	Reference	Reference
Middle tertile	3.4 (0.67-17.17)	.14
Highest tertile	65.3 (8.0-531.40)	<.001
Risk category^a^		
Lowest tertile of high-risk cases	Reference	Reference
Middle tertile of high-risk cases	0.5 (0.09-2.54)	.40
Highest tertile of high-risk cases	0.3 (0.056-1.75)	.19

a In multivariate analysis, only surgical volume significantly correlated with the presence of online information about neurodevelopment. Society of Thoracic Surgeons participation was excluded due to collinearity with surgical volume and surgical complexity. Programs were stratified into tertiles by ’15-’18 surgical volume and percent Society of Thoracic Surgeons–European Association for Cardio-Thoracic Surgery 4+5 cases. Highest annual surgical volume >270 cases; lowest <125 cases. Highest surgical complexity/risk of mortality >26.75% Society of Thoracic Surgeons–European Association for Cardio-Thoracic Surgery 4+5 cases; lowest <21.75% Society of Thoracic Surgeons–European Association for Cardio-Thoracic Surgery 4+5 cases.

### Qualitative Analysis

#### Sample Overview

The final dataset for qualitative analysis comprised 48 websites containing text, images, and videos. Data from one program were corrupted in the archival process, and one program had inadequate information for coding. Website content included information about neurodevelopment, the process for screening, and supports available at the individual institution, descriptions of research into the field, patient and family experiences, and links to external resources. The nature, amount, richness, and technicality of this information varied between programs.

#### Inductive Content Analysis

##### Three Cross-Cutting Functions

Taken in its entirety, the content and formatting of each website served three functions: (1) to convey information, (2) to form a connection with the reader, and (3) to establish the competence of the program. The information conveyed, the connection formed, and the emphasis on competence varied from site to site. Most websites described how CHD can alter children’s physical, mental, and social development. However, while some focused on how clinicians can help, others focused on the impact of these challenges on the child’s quality of life. Similarly, two primary strategies to connect with the reader emerged from the data: some websites used a formal, technical tone to establish authority, while others used a more collaborative style, sharing simplified, practical information. To establish competence, some websites referred to recommendations from organizations such as the Cardiac Neurodevelopmental Outcome Collaborative, American Heart Association, and the National Pediatric Cardiac Quality Improvement Collaborative, while others shared patient stories and testimonials [23].

##### Two Semantic Clusters

Differences across websites in terms of information conveyed, connection formed, emphasis on competence, and formatting formed the basis of two semantic clusters (Table 5). These two clusters parallel two previously described theories of adult learning and reflect the conceptualization of the institution as either a sage on the stage or a guide on the side [24]. The combined content and formatting used by websites in the first cluster emphasized the value of the services and expertise offered by the sponsoring institution, positioning their respective program as a sage on the stage. The features of the websites in the second cluster emphasized caregivers as partners in their children’s development, positioning their programs as guides on the side. Websites that clustered around the sage on the stage model focused on institutional expertise and communicated “who we are and what we do,” while those that more strongly reflected the guide on the side model focused on addressing children’s needs in partnership with parents or other caregivers and were more effective at sharing “what your child needs and how we can help.”

**Table 5.** Two semantic clusters were defined by the differential presentation and relative emphases of each website on the congenital cardiac surgical programs’ competence, connection to the patient, and services offered.

Function and format	Sage on the stage	Guide on the side
Convey information	Clinician focused: Centers on the clinician’s role Focuses on technical aspects of clinic visits Describes research output	Child focused: Centers on the child’s needs Focuses on practical details of clinic visits Describes toll of CHD^a^ on the entire family
Connect with the reader	Authoritative: Specialized language and jargon Technical images and illustrations Headshots of providers Emphasizes partnerships with other medical professionals Shares recommendations from professional organizations	Collaborative: Plain language Simplified images and illustrations Candid pictures of patients and providers Emphasizes partnership with children and their caregivers Shares patient stories Social media links Requests for donations Describes patient participation in research
Establish competence	Formal validation: Awards and certifications listed Scope and experience of program and providers emphasized	Anecdotal evidence: Patient testimonials
Format	Formal: Third person language Passive voice Serif fonts, taller than wide Few or no pictures or videos	Informal: First or second person language Active voice Sans-serif fonts, wider than tall Pictures and/or videos included

a CHD: congenital heart disease.

##### Sage on the Stage

Websites conforming to the sage on the stage model reflected an authoritative stance (Table 6). Introductory phrases tended to describe services offered by their programs, with a subsequent description of the neurodevelopmental challenges faced by children with CHD. In this way, they centered the institution, not the child. Institutional resources and expertise were emphasized by highlighting staffing, multidisciplinary collaboration, and specialization, as well as by using more technical images and formal typography.

Websites were more likely to use third-person and passive voice to describe both the challenges faced by children with cardiac concerns and the institution’s services. While this created a sense of impartiality, it also distanced the author from the reader and weakened the reader’s connection to the institution. Families and children were presented as the beneficiaries of partnerships between medical professionals and were described as the participants of, rather than collaborators in, research endeavors.

These features combined to create expert-centered websites, with children as the objects of their expertise, and characterized parents and other caregivers as assistants in their children’s care.

**Table 6.** Examples of features associated with the sage on the stage websites.

Function and format	Feature description	Examples
Convey informatin	Centers on the clinician’s role Focus on technical aspects of clinic visits Describes research output	The follow-up program, “tracks, monitors and manages the development of children with congenital heart disease who undergo surgical intervention as infants.” “Hammersmith Infant Screen and Bayley Developmental Scales.” “Conduct a range of translational and basic science research.” “Description of research of QI projects in cardiac neurodevelopment.”
Connect with the reader: authoritative	Picture: headshots of providers Images: technical Language: specialized/jargon Partnership: with medical professionals Appeals to authority	Cross-sectional image of a 3D rendering of a heart. “Impaired visual-spatial and visuo-motor skills.” “We partner with each family’s local pediatrician to develop a transparent approach to care.” “The American Heart Association and American Academy of Pediatrics recommend that all children with complex congenital heart disease receive regular developmental monitoring.”
Establish competence	Awards and certifications listed Scope and experience of program and providers emphasized	“US News and World Report Best Children’s Hospitals for Cardiology & Heart Surgery 2022‐2023 logo. American Nurses Credentialing Center Magnet Recognition logo. “As a world leader in pediatric cardiac care.” “We’re recognized across the country for establishing the first cardiac developmental follow-up program in the nation.” “The best care in the region for children with heart disease.” “Renowned experts.” “Revolutionary treatments.” “Team includes experts in physical and occupational therapy, speech therapy, a registered dietician, neuropsychologist, and pediatricians. A social worker is also available to support families.”
Format: formal	Third-person language Passive voice Font: serif, taller than wide Few or no pictures or videos	The heart center, “is dedicated to providing comprehensive care to all of our patients, including monitoring and treatment for any neurodevelopmental disabilities.”

##### Guide on the Side

Websites conforming to the guide on the side model addressed parents and other caregivers directly, using first- and second-person language, and described them as an active part of the team, collaborating with medical professionals to promote their child’s success (Table 7). They used plain language and defined technical terminology or jargon as it occurred.

**Table 7.** Examples of features associated with guide on the side websites.

Function and format	Feature description	Examples
Convey information	Centers on the child Focuses on practical details: insurance coverage, finding parking, paperwork involved, duration of visits, clinic phone numbers, links to online appointment desks, email addresses for coordinators Describes patient participation in research Toll of CHD^a^ on the whole family	“Your child’s first visit to the Cardiac Neurodevelopmental Clinic involves a thorough screening by providers from many different medical services. Each is an expert in a specific area of pediatric growth and development. This exam will take about 3 hours.” “Some families have found it helpful to bring snacks, drinks, toys or books to occupy their children during the appointment.” “Having a child with a serious heart condition…may leave you with a jumble of thoughts and feelings, including anger and grief….any and all thoughts or feelings you experience during this time are normal.”
Connect with the reader: collaborative	Pictures: candid pictures and videos of patients and providers Images: simplified Language: plain Partnership: with families Patient stories Social media links Requests for donations Research: invitation to participate	Image of a female health care provider in a medical setting sharing a teddy bear dressed in scrubs with a girl in a wheelchair. Image of a female nurse providing hands-on care to a female infant following open-heart surgery in an intensive care setting. “Neurodevelopment is a term that describes how our brains grow and form connections that direct our function and behaviors.” Links to Facebook (Meta), SnapChat (Meta), Twitter (formerly known as Twitter; X Corp), LinkedIn (Meta), Instagram (Meta), and YouTube (Google LLC) accounts. “We’ll be right by your side providing care.” “Together, we will decide if any other support or programs can help your child.” “If families and children are willing, voluntary participation in clinical research studies remains the cornerstone of the process.”
Establish competence	Patient testimonials	“Website section: “Stories to Inspire.” Stories about children at home with their families.
Format	First/second person language Active voice Font: sans-serif, wider than tall Pictures or videos included	“We look forward to partnering with you to help your child reach their fullest potential.” We will refer you to “specialists in your community who can help your baby reach key developmental milestones, such as walking and talking.”

a CHD: congenital heart disease.

Visual and verbal storytelling provided another mechanism for demonstrating mutual respect and building personal connections. Candid images of providers interacting with children in both inpatient and outpatient settings powerfully illustrated the relationships that can develop between children and their medical team. They also highlighted the approachability of these providers. Written accounts of children’s challenges and successes invited the reader to imagine what the best possible outcome for their child might look like and reminded them that they are not alone on their journey. Solicitations for donations or research participation similarly invited readers to partner with the institution to further the work of caring for children with CHD in developmentally appropriate ways.

These features combined to create websites that were patient-centered, respected caregiver authority, and promoted collaboration in the care of children with CHD.

##### Degree of Conformity

Few websites reflected a single pattern; rather, combinations of various elements on each website conformed more or less closely to one or the other. About half (25/48, 52%) of program websites conformed most closely to a guide on the side model, 40% (19/48) of them reflected a sage on the stage model, and 8% (4/48) of them had overlapping features of both. Websites from larger programs (≥20 cardiologists) were statistically more likely to follow a guide on the side model (20/28 vs 5/16, *χ*^2^_1_=6.7, *P*=.01). No other program- or state-level characteristics correlated with a program conforming to one vs the other learning model.

### Deductive Qualitative Analysis

#### Language Accessibility

All but two program websites were written above a 6th-grade reading level, with an average reading level between the 7th and 10th grade (Flesch-Kincaid mean 9.9, SD 2.4, Gunning Fog mean 9.0, SD 2.9, SMOG index mean 7.6, SD 1.9). Only two websites had information in a language other than English. Both offered information in Spanish.

#### Caregiver Utility

No more than one-third of websites included any one of the individual website features that most parents of children with CHD identified as useful. Only three program websites included all four features (Table 8).

**Table 8.** Inclusion of website features parents find useful.

Characteristic	Values, n (%)
A combination of words, pictures, and video	5 (10)
Descriptions of current research	16 (33)
Patient stories	16 (33)
Links to additional websites	16 (33)
All features	3 (6)

#### Transparency and Reliability

Of the 48 programs with codable data, the average JAMA benchmarks score was 1.5/4. Only four websites listed any authors, all of whom were authors for attached or embedded files, not for the main website. While nearly all programs included a copyright date on their website, only 1 program included information on when the website was most recently updated, with 3 other programs including the publication dates of attached or embedded files. A total of 15 programs cited sources for statements made on their website.

## Discussion

### Principal Findings

This study outlines the current paucity of information about neurodevelopment shared online by congenital cardiac surgical programs, identifies barriers to parents and other caregivers accessing that information, and identifies two website models corresponding to two models of adult learning.

### Comparison With Prior Work

#### Overview

Despite the potential neurodevelopmental impacts of CHD, children’s receipt of developmentally supportive care is inconsistent, and both short- and long-term outcomes remain variable [6,7,25-27]. Many factors associated with children receiving neurodevelopmental follow-up (hospital-initiated scheduling, antenatal diagnosis, and longer postoperative length of stay) suggest that caregiver understanding facilitates receipt of this care. Increased parent and caregiver education may improve follow-up with anticipated positive downstream impacts on patient outcomes [6]. Given caregivers’ reliance on online sources of health information and their documented use of congenital cardiac surgical program websites, these sites may be one venue to provide such education.

#### Limited Searchable Information About Cardiac Neurodevelopment

Parents and other caregivers of children with CHD report that online resources help them better understand their child’s diagnosis and the information from their child’s health care team. However, approximately a quarter of parents note that information about their child’s cardiac diagnosis is difficult to find online [11]. This study expands upon this parental observation, finding that only 39% (50/129) of congenital cardiac surgical programs in the United States include any information about one of the biggest contributors to these children’s quality of life, the neurodevelopmental sequelae of CHD, on their institutional websites. Due to poor search engine optimization, this information was difficult to find, with only 38 programs having information that could be found by a search of their institutional website and 47 having information that could be found via a Google search.

This lack of searchable information is troubling, as more than a third (39%) of parents and other caregivers report using websites, not their child’s health care provider, as their primary source of information about their child’s heart condition [12]. These numbers are higher for caregivers of patients on public insurance (45%) and caregivers with less than a high school education (48%). These children from socioeconomically disadvantaged backgrounds, whose parents and other caregivers rely more on online sources of information, are the same children who experience higher rates of mortality, longer lengths of stay, and higher medical costs following cardiac surgery [25-27]. This suggests a disproportionate impact of this missing information on those children already at risk for worse outcomes.

#### Language and Literacy Barriers

Parents and other caregivers face additional challenges accessing information, even when it is included on congenital cardiac surgical program websites. In this study, all but two program websites were written above a 6th-grade reading level, with an average reading level between the 7th and 10th grade. In addition, despite nearly 20% of US adults being foreign-born, many of whom speak English as a second language, in this study, only two congenital cardiac surgical program websites offered information in a language other than English [28].

Lower socioeconomic status, lower educational attainment, and speaking English as a second language are all associated with both lower literacy and lower health literacy [28,29]. Lower health literacy is associated with worse health outcomes in both children and adults [30-32]. No studies have explored the relationship between parent or other caregiver health literacy and outcomes following childhood cardiac surgery. However, children who live in lower-income neighborhoods, further from a specialized congenital cardiac surgical center, and who have public (as opposed to private) insurance have higher rates of mortality, longer lengths of stay, and higher medical costs following cardiac surgery [25-27]. This suggests that health literacy may be one of the mediators of health outcomes in these children and supports the American Heart Association recommendation that all health care communication be “accessible at any level of health literacy” [33].

Many medical organizations offer guidance on health care communication, for both print and online materials. The Joint Commission recommends that health care organizations know “the language needs and literacy levels of the population and community served” [34]. Practical recommendations can be found in the Health Literacy Universal Precautions Toolkit, created by the Agency for Healthcare Research and Quality [35]. Recommendations include having staff members skilled in assessing, selecting, and creating written materials that are easy to act on; obtaining patient feedback on written materials; routinely assessing whether written materials are easy to understand; and providing materials in the preferred language of the patients cared for.

The use of features that parents and other caregivers of children with CHD have previously identified as useful may also improve comprehension for parents and other caregivers with limited health literacy. These features include using a combination of words, pictures, and videos; sharing patient stories; and sharing summaries of recent research [11]. These features were infrequently found on the websites reviewed in this study, with each feature being found on no more than a third of the websites visited, and only three websites including all four features.

#### Sage on the Stage and Guide on the Side

Most surprisingly, we identified two semantic website clusters corresponding to two established adult educational models: the sage on the stage and the guide on the side. Those websites adhering to the sage on the stage model emphasized institutional expertise, reflecting an authoritarian and patriarchal stance toward patients and their loved ones. Those conforming more closely to the guide on the side model emphasized parental partnership and centered the patient and family experience with humility and empathy, reflecting a family-centered model of care.

Educational researchers continue to debate the relative merits of each model in promoting adult learning [24,36]. It remains to be seen if one, the other, or a combination of both models is most palatable to the parents and other caregivers of children with CHD and whether such differences impact parental follow-up adherence, parental stress, and child developmental outcomes.

### Recommendations for Congenital Cardiac Program Website Development

Congenital cardiac surgical program websites have the potential to improve parents’ and other caregivers’ understanding of their child’s unique developmental and mental health needs. This study reveals a lack of relevant information on surgical program websites, poor search engine optimization, language barriers, and a lack of features preferred by parents. To improve website effectiveness, there are several steps website developers can take (Textbox 1). These include the following: (1) including content about the developmental and mental health impacts of CHD; (2) performing internal and external search engine optimization; (3) removing language barriers by limiting jargon, using plain language, and providing information in their patients’ preferred languages; (4) including more features that caregivers find useful; and (5) including links to local resources. These simple changes could increase the impact of existing websites by helping caregivers recognize the anticipated neurodevelopmental sequelae of their child’s CHD and access services to support their child’s development.

Textbox 1. **Recommendations for website developers.**

1. Include information about neurodevelopment.
2. Perform internal and external search engine optimization (use metadata tags such as “cardiac neurodevelopment,” “development,” “ADHD,” and “neuropsychology”).
3. Write website content at a level that parents and patients can understand (sixth-grade reading level or below with active voice, no jargon, short paragraphs, and simple illustrations).
4. Provide information in the languages spoken by patients and their caregivers.
5. Include website features that parents of children with CHD find useful (a combination of words, pictures, and videos; descriptions of current research; patient stories; and links to additional resources).
6. Include links to local, community-based resources.

Additional improvement could be realized by institutional application of the principles underpinning the guide on the side model—empathy, humility, and collaboration—through the process of human-centered design [37]. By seeking out and incorporating parent and other caregiver perspectives at all stages of website development, institutions could identify the preferred model of content delivery (sage on the stage vs guide on the side) for patients and families in their catchment area and develop strategies for cognitive alignment. Centering the parents and other caregivers of children with CHD will allow for optimized delivery of accessible, impactful, and usable information.

### Limitations

There were several methodological limitations. While this study intended to include a total population sample of all programs performing congenital cardiac surgery in the United States, 1 surgical program was not included in the Congenital Cardiology Today directory for 2021‐2022, and data for 1 program were lost during the archival process. While hospital catchment areas vary in size, from portions of a city to multiple states, this study compared congenital cardiac surgical programs to state-level sociodemographic data and may have missed local or regional factors. In addition, STS (Society of Thoracic Surgeons) Congenital Cardiac Public Reporting Data from 2015‐2018 was used instead of 2019‐2023 data. More recent data encompasses surgical volumes and outcomes during the height of the COVID-19 pandemic, which varied substantially from surgical program operations both before and after the pandemic, which would have skewed the data [38,39].

All web searches were conducted in English without the use of incognito mode, or clearance of caches and cookies, potentially influencing search results and leading to either missed or enhanced identification of neurodevelopmental information due to the heavy personalization of search engine algorithms based on browsing history. While many congenital cardiac surgical programs have a social media presence, this study did not evaluate their social media. This was an intentional choice, as more caregivers of children with CHD report using websites than social media (72%‐92% vs 20%, respectively) to learn about their child’s health and development [11,12]. Finally, the choice of inductive content analysis facilitated analysis of what was present on program websites, but not an analysis of potentially useful features that were absent, such as links to or descriptions of how to access local, community-based early intervention programs.

### Next Steps

This analysis of information about neurodevelopment on the websites of congenital cardiac surgical programs invites future inquiry into both the topic at hand and the broader application of our findings to other pediatric medical subspecialties. First, what are parents’ and other caregivers’ impressions of available information, and how does the way in which the information is presented (guide on the side vs sage on the stage models) impact follow-up adherence, caregiver stress, and children’s outcomes? Second, how does health literacy mediate disparities in health and neurodevelopmental outcomes in children with CHD? Third, what is the quality of other online sources of information (nonprofit and governmental websites, social media sites, and parent support groups) about the neurodevelopmental outcomes of children with CHD? Finally, do these findings generalize to other areas of pediatric medical subspecialty care, and can we leverage our presentation of online information to improve children’s outcomes across multiple domains?

### Conclusions

CHD is a complex, lifelong condition that creates ongoing informational needs for impacted patients and their parents and other caregivers. Neurodevelopmental impairments are the number one morbidity of CHD. Public-facing information about cardiac neurodevelopment on the websites of congenital cardiac surgical programs is sparse, with high-volume surgical programs being the most likely to have any information at all. For websites that do include this information, linguistic factors and poor search engine optimization create additional barriers for parents and other caregivers to access this information. Websites reflect two models of adult learning: the guide on the side, which emphasizes caregivers as key stakeholders and partners in their children’s development, and the sage on the stage, which emphasizes the value of the services and expertise offered by the sponsoring institution. Future studies should evaluate which, if either, model is preferred by caregivers or more strongly influences caregiver behaviors and patient outcomes. This would allow for the development of content that promotes caregivers’ pursuit of optimal neurodevelopmental supports for their children and may have application to other areas of pediatric medical subspecialty care as well.

## Abbreviations

CHD: congenital heart disease
JAMA: Journal of the American Medical Association
SMOG: Simplified Measure of Gobbledygook
STS: Society of Thoracic Surgeons
